# Surgical Resection for a Second Primary Lung Cancer Originating Close to the Initial Surgical Margin for Lung Squamous Cell Carcinoma

**DOI:** 10.1155/2015/462193

**Published:** 2015-03-10

**Authors:** Seijiro Sato, Terumoto Koike, Takehisa Hashimoto, Masanori Tsuchida

**Affiliations:** Division of Thoracic and Cardiovascular Surgery, Niigata University Graduate School of Medical and Dental Sciences, 1-757 Asahimachi-dori, Chuo-ku, Niigata-shi, Niigata 951-8510, Japan

## Abstract

Few reports have described surgical resection for second primary lung cancers originating close to the initial surgical margin for lung cancer. A 64-year-old man had undergone left segmentectomy with lymph node dissection for small peripheral squamous cell lung cancer using video-assisted thoracic surgery, with pathology confirming a small tumor 12 mm in diameter identified about 3 cm from the surgical margin. Eighteen months after initial surgery, computed tomography revealed a 30 mm pulmonary nodule close to the initial surgical margin in the residual left upper lobe and the serum level of carcinoembryonic antigen was found to be increased. Local recurrence on the staple-line of the surgical margin was suspected, and completion left upper lobectomy was performed. Histological examination identified not only a squamous cell carcinoma component but also a small cell carcinoma component. The immunohistochemical staining pattern of the second tumor differed from that of the initial resected lung squamous cell carcinoma. The final pathological diagnosis was a second primary tumor with mixed small cell carcinoma and squamous cell carcinoma histology.

## 1. Introduction

In recent times, small peripheral malignant lung tumor has increasingly been treated by limited resection using video-assisted thoracic surgery (VATS) to minimize the volume of lung resected and the size of the thoracotomy incision [1]. Staplers have routinely been used and various complications of the surgical margins have been reported [2–4] with this increasing use. A new lesion originating close to the initial surgical margin during postoperative follow-up is one such complication. Principal differential diagnoses for such lesions include local recurrence of the initial lung malignancy, nontuberculous mycobacterial infection or fungal infection caused by nonanatomical stapling, and foreign-body granuloma. However, in addition to these diseases, the possibility of a second primary lung cancer should be considered. To the best of our knowledge, only one previous report [5] has described a second primary lung cancer originating close to the initial surgical margin. We report herein a case of a second primary lung cancer originating close to the initial surgical margin for a previous lung squamous cell carcinoma and treated by surgical segmentectomy.

## 2. Case Presentation

**Figure 1:**
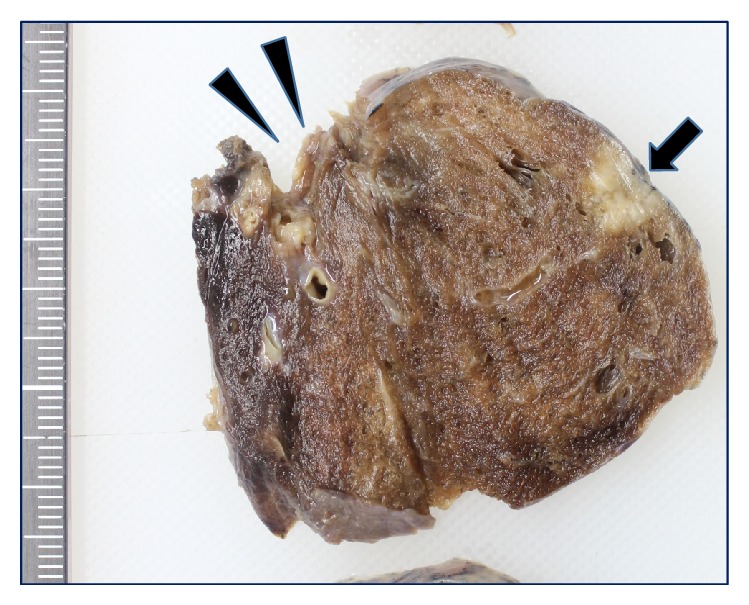
The tumor (arrow) measuring 13 mm is about 3 cm away from the surgical margin (arrowhead).

**Figure 2:**
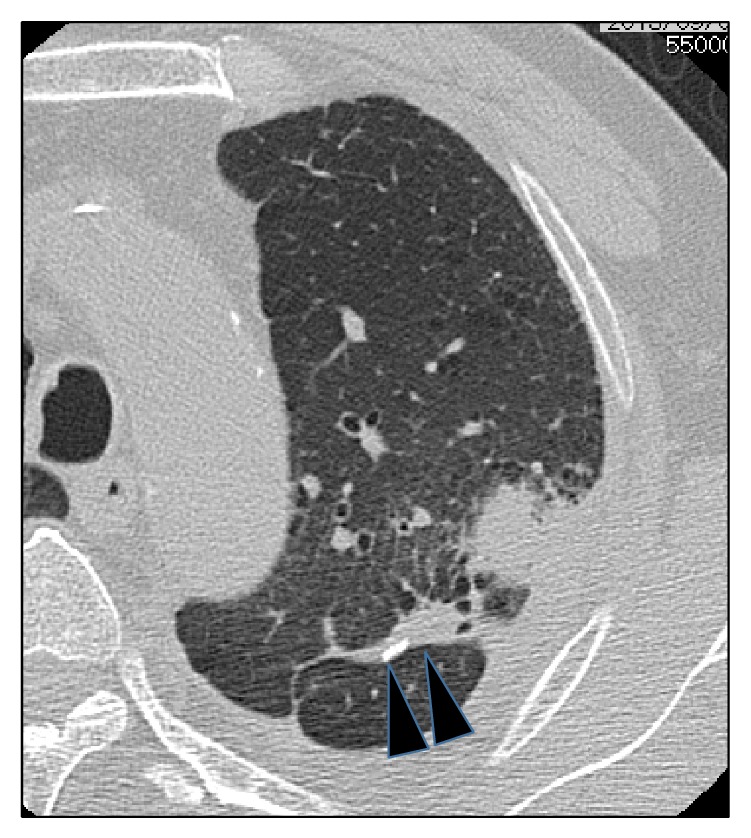
Chest CT reveals a 30 mm pulmonary nodule close to the initial surgical margin (arrowhead).

A 64-year-old man underwent segmentectomy with lymph node dissection for lung cancer of left segments 1 + 2 in March 2012. Pathologically, the tumor was diagnosed as a moderately differentiated squamous cell carcinoma, measuring 12 × 8 mm (pT1aN0M0). Immunohistochemical staining showed positive expression of CK5/6 and p63 and negative expression of thyroid transcription factor 1 (TTF-1), CD56, chromogranin A (CGA), and synaptophysin. The tumor was about 3 cm from the surgical margin and no residual cancer cells were identified (Figure 1). In October 2013, serum levels of carcinoembryonic antigen (CEA) were found to be elevated. Computed tomography (CT) revealed a 30 mm pulmonary nodule close to the initial surgical margin (Figure 2). Positron emission tomography (PET) with ^18^F-fluorodeoxyglucose (FDG) showed uptake by the tumor, but no region of uptake other than the equivocal uptake area. In November 2013, the patient was admitted on suspicion of local recurrence along the staple-line of the surgical margin. He had a 44-year history of smoking 1 pack/day until 2 years earlier. Physical examination yielded normal results. Laboratory data showed that the serum level of CEA was 12.1 ng/mL (normal, <4.3 ng/mL), and pro-gastrin-releasing peptide (ProGRP) level was 134 pg/mL (normal, <81 pg/mL). Pulmonary function testing showed normal result. Reoperation was performed, with intraoperative rapid diagnosis suggesting squamous cell carcinoma, and completion left upper lobectomy was performed.

**Figure 3:**
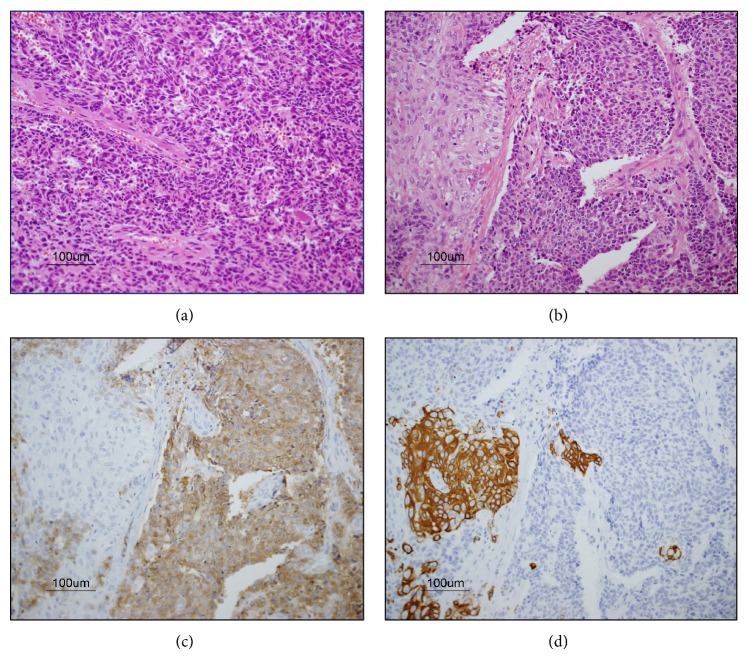
Histopathological examination shows that the tumor comprises a main small cell carcinoma component (a) and a smaller squamous cell carcinoma component (b). Immunohistochemically, tumor cells of the small cell carcinoma component show positive staining for CD56 (c) but negative staining for CK5/6 (d). By contrast, cells of squamous cell carcinoma component were positive for CK5/6 (d) but negative for CD56 (c).

Macroscopically, the tumor was a solid, whitish mass measuring 32 × 25 mm and showing partial necrosis. Histological examination identified a main small cell carcinoma component with a high nuclear-cytoplasmic ratio, unclear nucleoli, and fine chromatin (Figure 3(a)) and a smaller squamous cell carcinoma component (Figure 3(b)). Immunohistochemically, tumor cells of the small cell carcinoma component showed positive staining for thyroid transcription factor 1 (TTF-1), chromogranin A (CGA), synaptophysin, and CD56 (Figure 3(c)) but negative staining for p63 and CK5/6 (Figure 3(d)). By contrast, cells of squamous cell carcinoma component were positive for p63 and CK5/6 (Figure 3(d)) but negative for TTF-1, CGA, synaptophysin, and CD56 (Figure 3(c)). The staining pattern of the second tumor differed from that of the squamous cell carcinoma resected from the lung previously. For these reasons, pathological diagnosis was a second primary combining small cell lung carcinoma and squamous cell carcinoma and intrapulmonary lymph node metastasis (pT2aN1M0). The postoperative course was uneventful, and the patient received chemotherapy for small cell lung cancer.

## 3. Discussion

Reports of lung tumors close to the initial surgical margin of a resected lung cancer resection are gradually increasing as reduction surgeries and staplers for dissection of the intersegmental plane see greater use. One differential diagnosis is surgical-margin recurrence after lung cancer resection. Another is an infectious mass occurring after ventilatory impairment and blood flow obstruction in the lung tissue from stapling result in the development of an infection from pathogens that were present preoperatively [3]. A third possibility is foreign-body granuloma, a noninfectious mass resulting from a foreign-body (antigen) response activating T-cells and macrophages that cause epithelioid cells and giant cells to emerge [5].

In making a differential diagnosis from imaging, CT often reveals a noncancerous lesion with a linear margin, calcification, diffuse shadows, and no involvement of blood vessels and bronchial tubes [6, 7]. Recent reports have indicated that fluorodeoxyglucose- (FDG-) PET is not particularly informative, with a diagnostic sensitivity for malignancy of 63%, specificity of 56%, and a proper diagnosis rate of 60% for nodes ≤30 mm and a threshold maximum standardized uptake value (SUVmax) of 2.5 [8].

Including the present report, 13 cases of lung masses close to the initial surgical margin have been reported in Japan (Table 1) [2–5, 9–16]. However, many represented nontuberculous mycobacteria or foreign-body granuloma. Diagnosis was obtained in only one [5] of the five cases in which preoperative diagnosis was attempted, indicating that preoperative diagnosis is very difficult. Only four patients underwent PET-CT preoperatively, and the SUVmax for the infectious mass [3] was high, at 4.59, suggesting difficulty in using this approach to identify malignancy. Only two of the 13 patients were diagnosed with a second primary lung cancer, and multiple primary lung cancers are extremely rare.

At present, the adequacy of segmentectomy for lung cancers ≤2 cm with radiologically pure solid lesion remains controversial. The results of ongoing prospective randomized trials of lobar versus sublobar resection in patients with small peripheral non-small-cell lung cancers, such as JCOG 0802/WJOG 4607L [17], are eagerly awaited. We performed segmentectomy for the lung cancer ≤2 cm with pure solid component at initial surgery, because no involvement of hilar lymph nodes was evident intraoperatively.

In the present case, there were several reasons why we diagnosed the tumor as a second primary lung cancer rather than local recurrence along the staple-line of the surgical margin. First, from the perspective of the histological findings, including immunohistochemical staining, the lung cancer specimen from the initial surgery differed from the second primary specimen. The pathological diagnosis was squamous cell lung cancer in the initial surgery, compared to combined small cell lung cancer and squamous cell lung cancer in the second surgery. Second, a few reports [18, 19] have described cases in which EGFR-mutant non-small-cell lung carcinoma has acquired resistance to EGFR-TKI therapy through transformation to small cell lung cancer. However, the patient in this case had not received any postoperative treatment for cancer, including molecular targeted therapy, and local recurrence of the initial squamous cell carcinoma with transformation to small cell lung cancer was deemed highly unlikely. Third, the second primary tumor was about 3 cm from the initial resection stump.

## 4. Conclusion

Cases of new nodule originating close to the initial surgical margin seem to have increased in frequency, possibly because selected limited resection and the use of staplers for dissection of the intersegmental plane have increased. In addition to local recurrence, infectious nodules, and foreign-body granuloma, the possibility of a second primary lung cancer should be considered in such cases.

## Figures and Tables

**Table 1:** Reports of new nodules originating close to the initial surgical margin in Japan.

Author	Tanaka et al. [9]	Tomita et al. [2]	Kono et al. [10]	Katsura et al. [4]	Furukawa et al. [11]	Matsuoka et al. [12]	Ohtsuka et al. [13]	Murakami et al. [3]	Matsuoka et al. [14]	Tempaku et al. [15]	Motono et al. [16]	Kitahara et al. [5]	Our patient

Year	2003	2003	2005	2005	2007	2007	2008	2009	2011	2012	2012	2013	2015

Age	50	74	60	76	57	62	69	72	60	59	64	77	66

Sex	F	M	M	F	F	F	F	F	M	F	M	M	M

Initial surgical procedure	Segmentectomy	Wedge	Segmentectomy	Wedge	Segmentectomy	Lobectomy + segmentectomy	Wedge	Lobectomy	Wedge	Wedge	Wedge	Wedge	Segmentectomy

PET-CT	N/A	N/A	N/A	N/A	N/A	N/A	N/A	SUVmax 4.59	SUVmax 1	SUVmax 1.9	N/A	N/A	Strong accumulation

Preoperative diagnosis	FBS: negative	N/A	FBS: negative	N/A	FBS: negative	CTNB: negative	N/A	N/A	N/A	N/A	N/A	FBS: adenocarcinoma	N/A

Pathology of secondary nodule	Pulmonary tuberculosis	Pulmonary foreign body granuloma	Mycobacterial granuloma	Pulmonary suture granuloma with *Aspergillus *	Mycobacterial granuloma	Mycobacterial granuloma	Pulmonary foreign body granuloma	Mycobacterial granuloma	Local recurrence	Pulmonary foreign body granuloma	Pulmonary foreign body granuloma	Second primary lung cancer	Second primary lung cancer

Disease-free interval	60 m	24 m	28 m	24 m	48 m	51 m	57 m	96 m	56 m	60 m	7 m	72 m	18m

F: female; M: male; N/A: not available; FBS: fiber bronchoscopy; CTNB: CT-guided needle lung biopsy.
